# Hybrid endoscopic stricturotomy and balloon dilation of ascending colon stricture with cecal fecaliths in Crohn’s disease guided by preprocedural intestinal ultrasound

**DOI:** 10.1016/j.vgie.2025.05.005

**Published:** 2025-05-31

**Authors:** Partha Pal, Mohammad Abdul Mateen, Rajesh Gupta, Manu Tandan, D. Nageshwar Reddy

**Affiliations:** 1 Medical Gastroenterology, Asian Institute of Gastroenterology, Hyderabad, India; 2 Diagnostic Ultrasound and Radiology, Asian Institute of Gastroenterology, Hyderabad, India

## Abstract

**Background and Aims:**

Endotherapy for Crohn’s disease (CD) strictures has evolved as a minimally invasive alternative to surgery. Hybrid techniques combining endoscopic stricturotomy (ES) and balloon dilation (EBD) can improve outcomes. Intestinal ultrasound (IUS) has emerged as a point-of-care tool for stricture assessment. We present a case using preprocedural IUS-guided hybrid ES and EBD to manage an ascending colon stricture with proximal fecaliths in CD.

**Methods:**

A 47-year-old man with ileocolonic CD (Montreal classification A2, L3, B2) receiving adalimumab and azathioprine presented with recurrent obstructive symptoms. IUS identified a short, predominantly fibrotic, ascending colon stricture. Earlier computed tomography enterography 6 months back ruled out additional strictures. On colonoscopy, ES was performed using an insulated-tip knife, followed by EBD up to 12 mm. Redundant mucosa was excised, and minor bleeding controlled. The stricture was successfully traversed, and fecaliths were extracted.

**Results:**

The procedure was uneventful, and the patient was discharged the next day after adalimumab escalation. At 9-month follow-up, he remained symptom-free.

**Conclusions:**

Hybrid ES and EBD guided by preprocedural IUS offer an effective multimodal approach for CD strictures, potentially delaying or avoiding surgery. Further studies are warranted to validate the long-term role of IUS in stricture management.

## Case description

**Figure 1:**
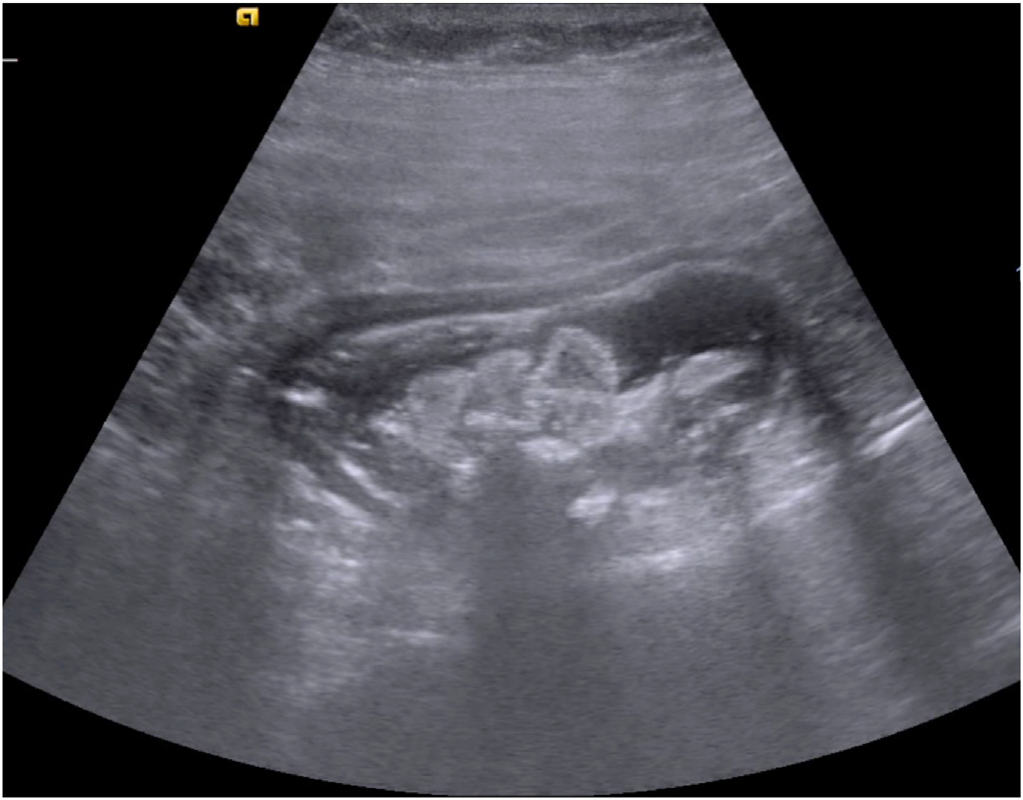
Intestinal ultrasound demonstrating an ascending colon stricture with upstream fecaliths, confirming luminal narrowing and prestenotic dilation.

**Figure 2:**
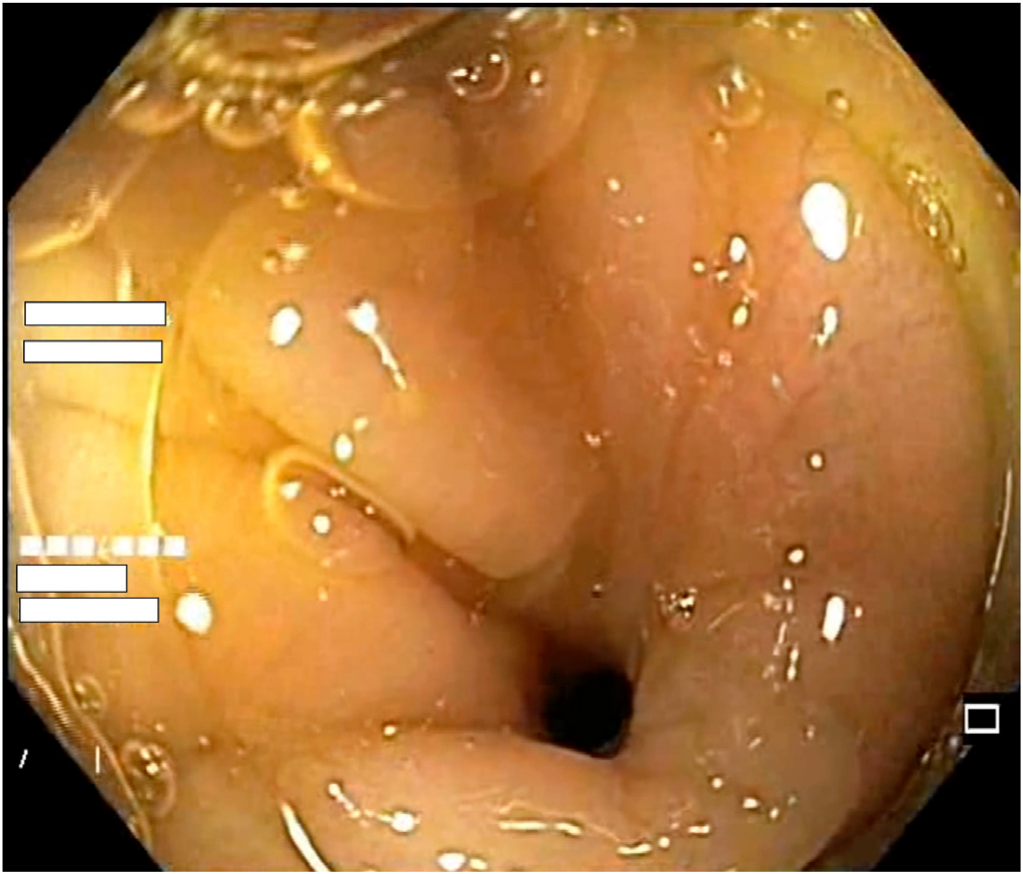
Colonoscopic view showing a short, tight fibrotic stricture in the ascending colon, impassable with a standard adult colonoscope.

**Figure 3:**
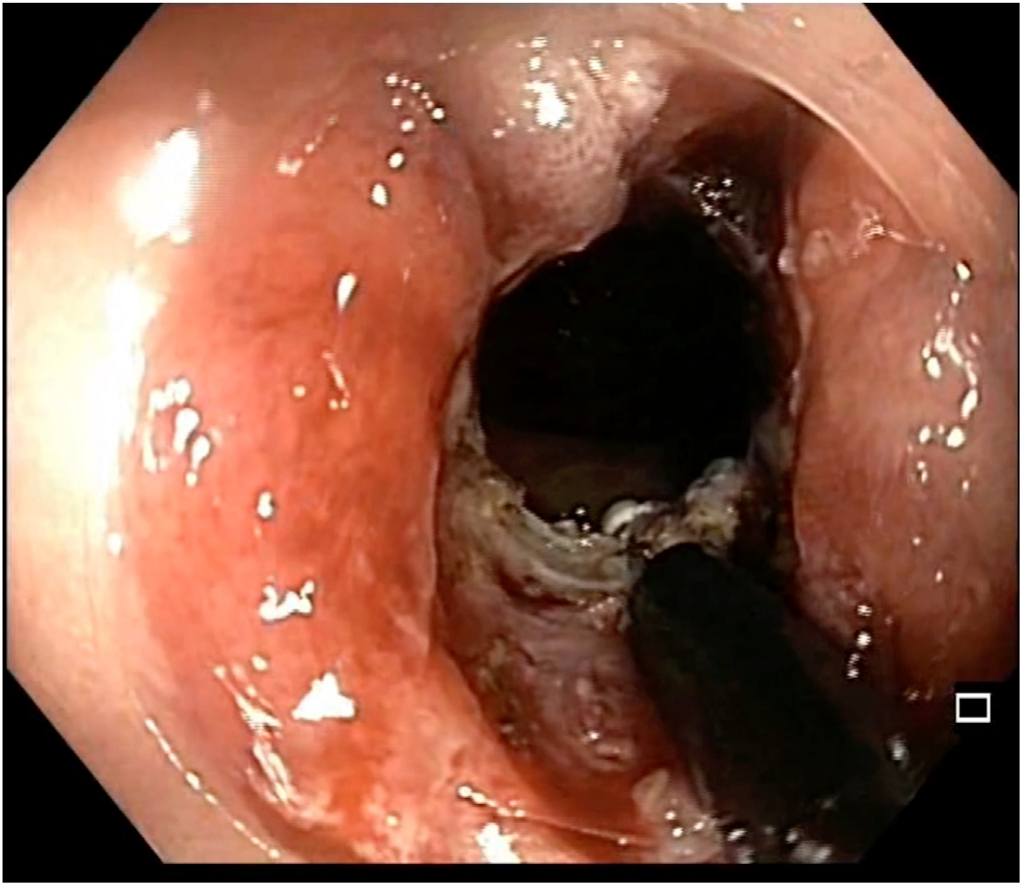
Endoscopic stricturotomy being performed using an insulated-tip knife with electrocautery settings for controlled mucosal incision.

**Figure 4:**
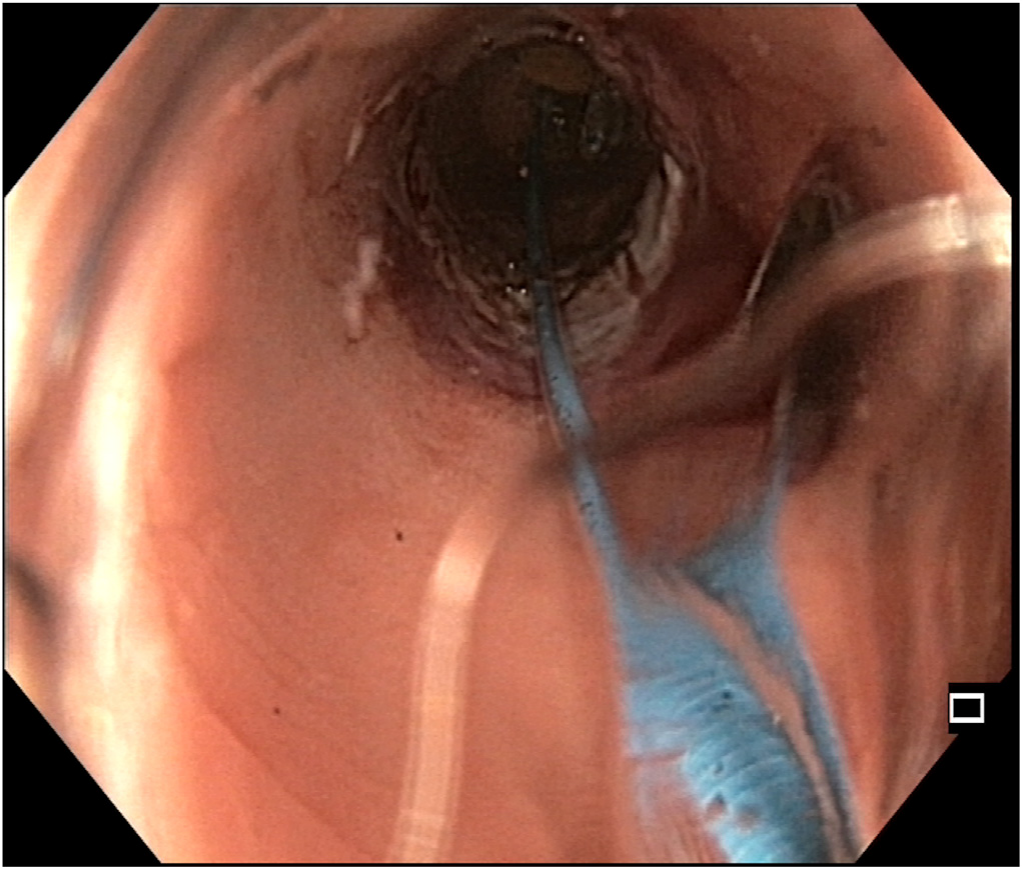
Balloon dilation of the stricture using a controlled radial expansion balloon, expanding the luminal diameter up to 12 mm to relieve obstruction.

**Figure 5:**
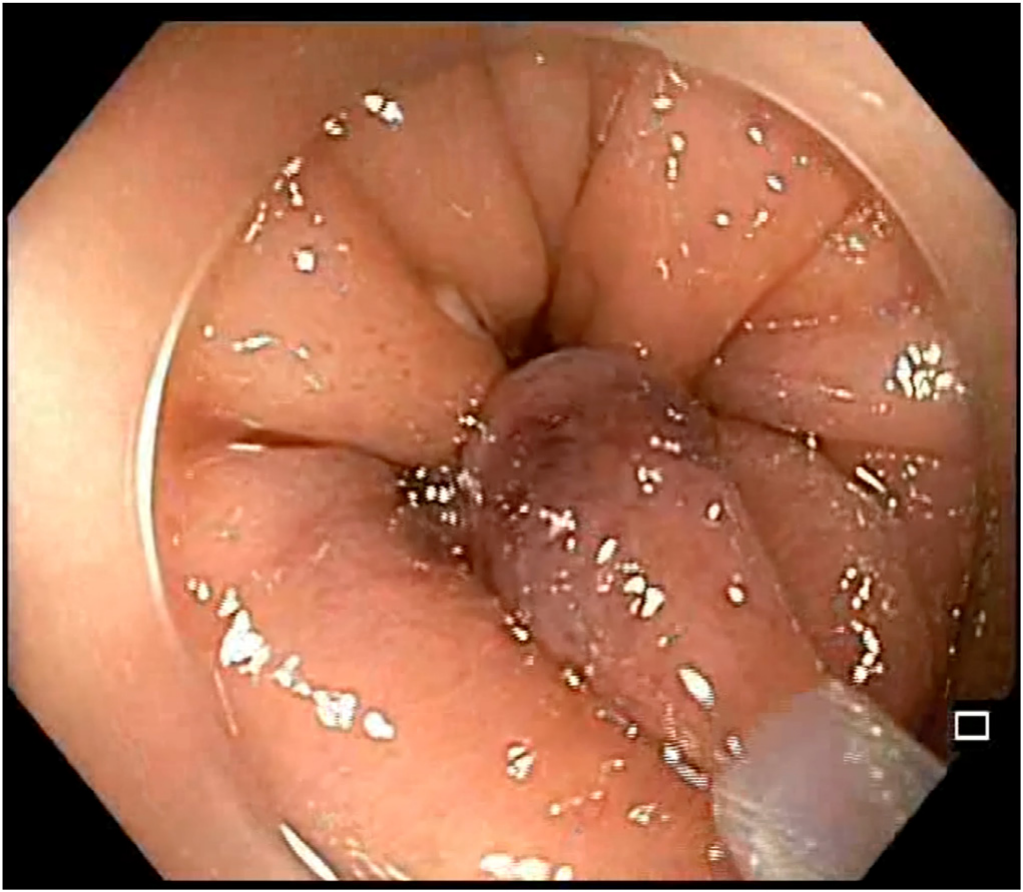
Snare excision of redundant mucosa around the stricture to improve visualization and optimize postprocedural luminal patency.

**Figure 6:**
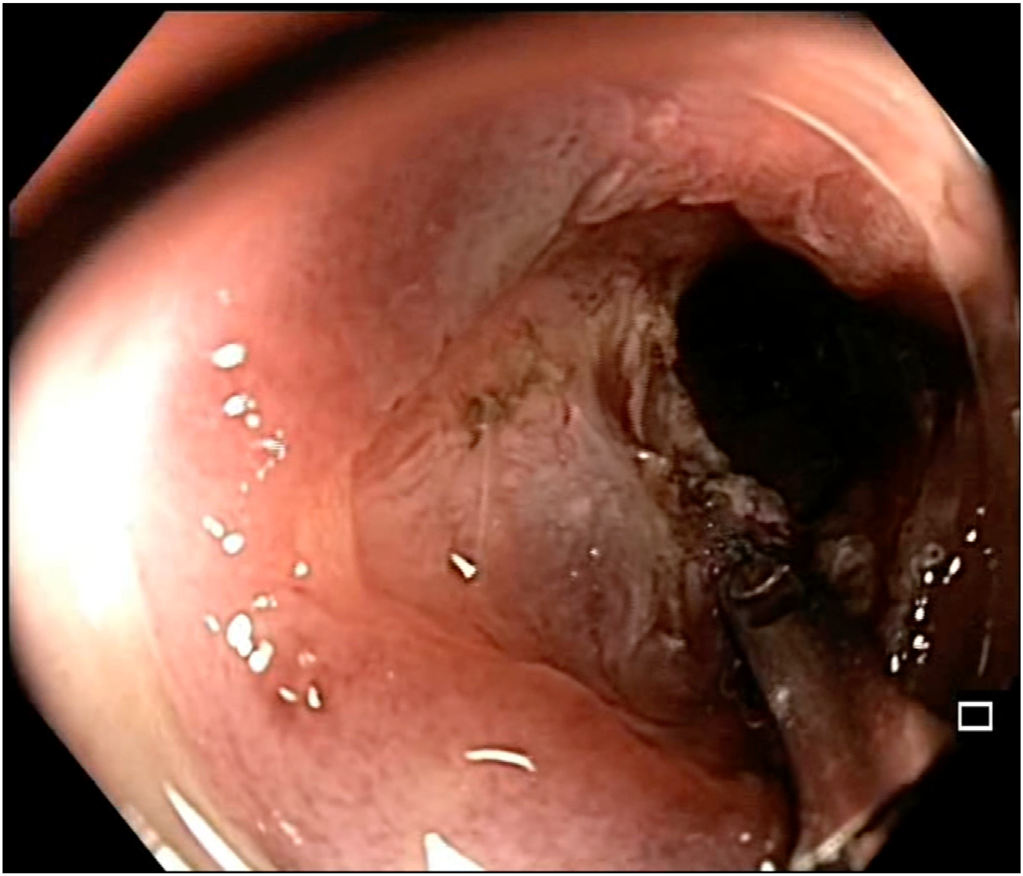
Minor bleeding during stricturotomy successfully controlled using a Coagrasper (Olympus Medical Systems, Tokyo, Japan).

**Figure 7:**
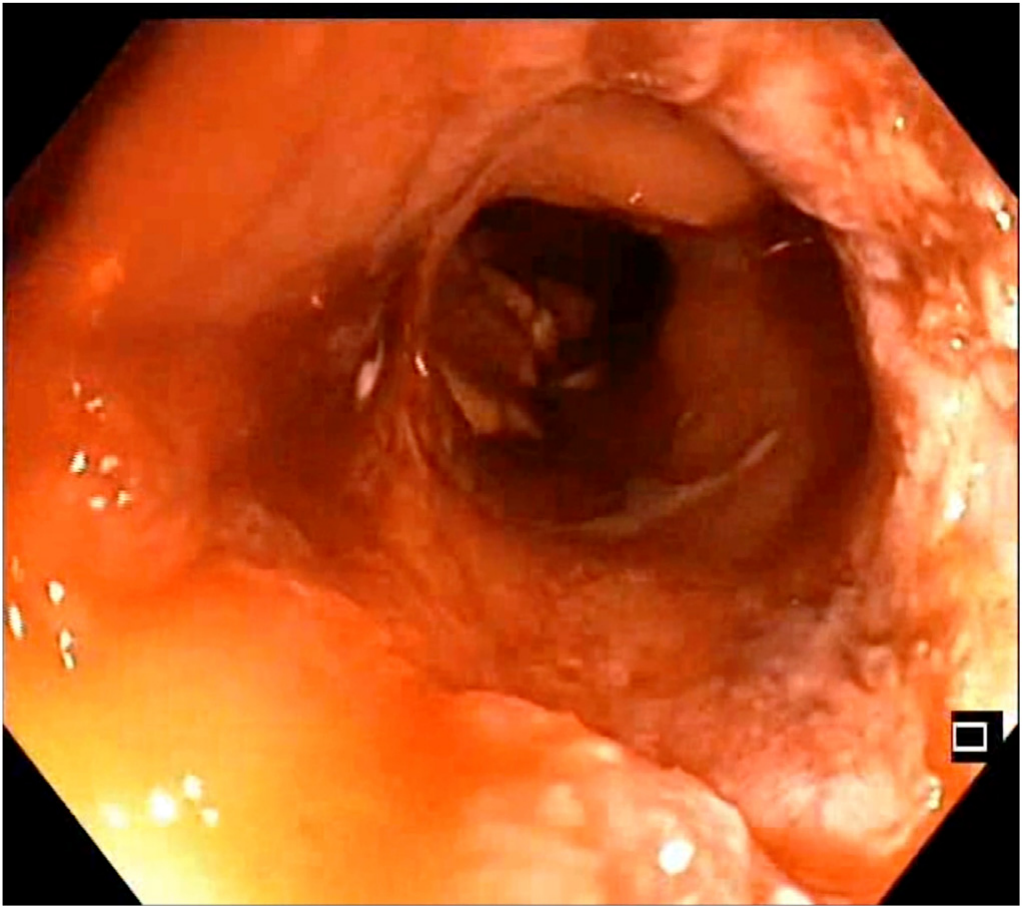
Poststricturotomy and dilation view, demonstrating successful passage of a pediatric colonoscope through the previously obstructed stricture.

**Figure 8:**
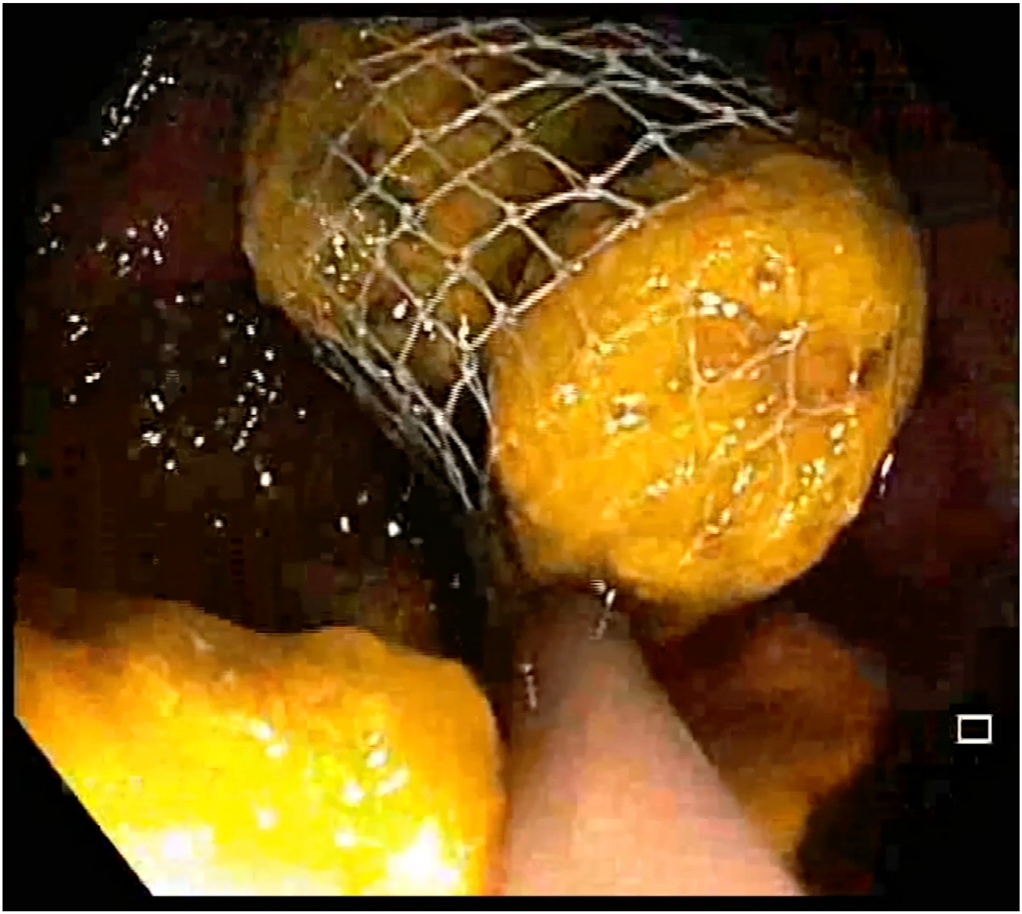
Fecaliths in the cecum retrieved using a Roth Net (US Endoscopy, Mentor, Ohio, USA) to prevent further obstruction and ensure complete stricture resolution.

**Figure 9:**
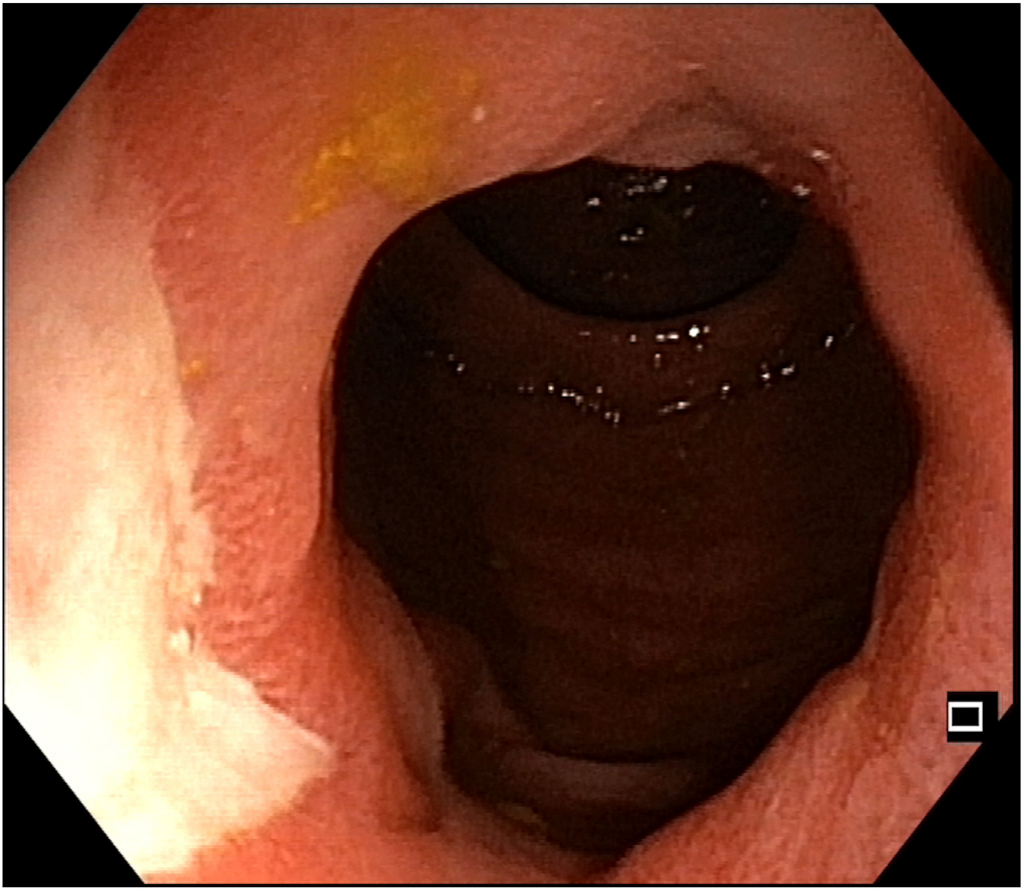
Ulceration observed near the deformed ileocecal valve, likely due to chronic inflammation, necessitating escalation of adalimumab therapy.

A 47-year-old man with ileocolonic Crohn’s disease (CD) (Montreal classification A2, L3, B2) receiving adalimumab and azathioprine presented with recurrent obstructive symptoms. He had undergone computed tomography enterography 6 months earlier that demonstrated an isolated ascending colon narrowing (Video 1, available online at www.videogie.org). However, he was relatively asymptomatic and opted for continuing medical therapy alone. Point-of-care intestinal ultrasound (IUS) revealed an ascending colon stricture with proximal fecaliths (Fig. 1). IUS demonstrated a stricture wall thickness of 5.5 mm, residual lumen of approximately 3 mm, focal loss of wall stratification, and absence of significant hypervascularity (modified Limberg score 1), indicating a predominantly fibrotic nature. Colonoscopy showed a tight, short fibrotic stricture in the ascending colon (Fig. 2). Based on the patient's preference for endoscopic therapy and after shared decision-making involving surgical consultation and a preanesthetic evaluation to manage any inadvertent adverse events requiring surgery, an endoscopic stricturotomy (ES) was performed using an insulated-tip knife (ITknife; Olympus Medical Systems, Tokyo, Japan) with a therapeutic colonoscope (Olympus CF-H180AL/I; Olympus Medical Systems) and distal cap (Fig. 3) using electrocautery settings (ENDO CUT I 3-1-3, VIO 300D; Erbe Elektromedizin GmbH, Tübingen, Germany), followed by dilation using a controlled radial expansion balloon (Boston Scientific, Marlborough, Mass, USA) up to 12 mm (Fig. 4). Redundant mucosa around the stricture was excised with a snare for better visualization (Fig. 5). Minor bleeding during the procedure was controlled with a Coagrasper (Olympus Medical Systems) (Fig. 6). The stricture was successfully traversed with a pediatric colonoscope (Olympus PCF-PH170L; Olympus Medical Systems) after combined ES and dilation (Fig. 7). Fecaliths in the cecum were retrieved using a Roth Net (US Endoscopy, Mentor, Ohio, USA) (Fig. 8). There was an ulcer near the deformed ileocecal valve (Fig. 9).

The procedure was completed in 2 hours and 20 minutes without adverse events, and the patient was discharged the following day with escalation of adalimumab to weekly dosing. At 9-month follow-up, he remains symptom-free (Video 1). The patient maintained steroid-free clinical remission during follow-up, without the need for additional biologics.

This case highlights the efficacy of integrating ES, endoscopic balloon dilation (EBD), and IUS in the management of complex CD adverse events.^1^ By combining these advanced endoscopic and imaging techniques, we were able to successfully alleviate obstructive symptoms, avoid surgical intervention, and preserve bowel integrity. The use of IUS as a preprocedural adjunct provided precise localization and characterization of the stricture, offering a noninvasive yet highly accurate diagnostic modality, avoiding repeated cross-sectional imaging.^2^ Precise electrocautery in ES combined with complementary EBD techniques could improve symptom control and delay the need for surgical interventions. Importantly, ES has been associated with longer reintervention-free survival and lower perforation risk (albeit with a higher bleeding risk) than EBD alone, underscoring the value of a hybrid approach that combines the advantages of both techniques while mitigating their individual limitations.^3^^,^^4^ Although mucosectomy theoretically carries a risk of promoting localized fibrosis and potential recurrence, in this case, redundant mucosal excision was performed judiciously and limited to improve visualization and lumen patency, minimizing deep-tissue injury.

By tailoring interventions to individual anatomical, disease-specific challenges, and incorporating patient preference for endoscopic therapy through shared decision-making, this approach reinforces the growing trend toward personalized care in inflammatory bowel disease.^1^^,^^5^ Further studies evaluating the long-term outcomes of such hybrid techniques and the role of IUS-guided endoscopic therapy in broader clinical contexts will be crucial in optimizing management strategies for CD.

## Patient consent

Written informed consent was obtained from the patient for the publication of the information and imaging.

## Disclosure

All other authors disclosed no financial relationships

## Supplementary data

Video 1 Hybrid endoscopic stricturotomy and balloon dilation of ascending colon stricture with cecal fecaliths in Crohn disease guided by intestinal ultrasound. This video demonstrates a stepwise hybrid approach using intestinal ultrasound (IUS)–guided endoscopic stricturotomy and balloon dilation for a fibrotic ascending colon stricture with upstream cecal fecaliths in Crohn disease. The procedure includes IUS localization, stricturotomy with electrocautery, balloon dilation up to 12 mm, and snare excision of redundant mucosa. Minor bleeding is controlled with a Coagrasper. After the procedure, a pediatric colonoscope traverses the stricture, and cecal fecaliths are retrieved with a Roth Net, ensuring successful obstruction relief and luminal patency.

## References

[1] Shen B., Kochhar G., Navaneethan U. (2020). Practical guidelines on endoscopic treatment for Crohn's disease strictures: a consensus statement from the Global Interventional Inflammatory Bowel Disease Group. Lancet Gastroenterol Hepatol.

[2] Pal P., Mateen M., Pooja K. (2024). Gastrointestinal: leveraging intestinal ultrasound to guide endoscopic closure of the internal opening of a vesico-sigmoid fistula in ileo-colonic Crohn's disease. J Gastroenterol Hepatol.

[3] Lan N., Shen B. (2018). Endoscopic stricturotomy versus balloon dilation in the treatment of anastomotic strictures in Crohn's disease. Inflamm Bowel Dis.

[4] Nobel Y.R., Shen B. (2020). Combined endoscopic stricturotomy and balloon dilation of strictureplasty site stricture in Jejunal Crohn's disease. ACG Case Rep J.

[5] Pal P., Nabi Z., Ramchandani M. (2025). Endoscopic stricturotomy (standalone, hybrid and graded) for refractory inflammatory bowel disease strictures: case series with technical review (with videos). Indian J Gastroenterol.

